# Characterization of Immune Infiltrating Cells in Bladder Urothelial Carcinoma and Its Clinical Significance

**DOI:** 10.1002/cam4.71737

**Published:** 2026-03-24

**Authors:** Xin Yan, Xiaoyu Tang, Linfa Guo, Ziqi He, Shaoxi Liu, Shaojie Wu, Zhilong Li, Tong‐Zu Liu, Hang Zheng, Weibing Zhang, Sheng Li

**Affiliations:** ^1^ Department of Urology Cancer Precision Diagnosis and Treatment and Translational Medicine Hubei Engineering Research Center, Zhongnan Hospital of Wuhan University Wuhan Hubei China; ^2^ Department of Urology Institute of Urology, West China Hospital, Sichuan University Chengdu Sichuan China; ^3^ Department of Urology Renmin Hospital of Wuhan University wuhan Hubei China; ^4^ College of Life Sciences and Health Wuhan University of Science and Technology Wuhan Hubei China

**Keywords:** bladder urothelial carcinoma, drugs, immune cell, immunotherapy, prognosis

## Abstract

**Background:**

Immune cells have been linked to the initiation and progression of tumors, and their presence is often used to predict disease prognosis. However, when it comes to bladder urothelial carcinoma (BLCA), there has not been a comprehensive investigation into the function and prognostic value of different immune cell types.

**Methods:**

We integrated data from more than 2300 BLCA patients across 14 public datasets. Then we analyzed the quantity of 170 different immune cell signatures using the ssGSEA algorithm. Through meta‐analysis and univariable Cox analysis, we identified prognosis‐associated immune cells and established an immune cell related prognostic signature (IRPS). We then conducted survival analyses to observe the differences in survival across different IRPS‐risk groups. Furthermore, based on the DEGs associated with IRPS, we screened for potential targeted therapeutic agents. Finally, we integrated IRPS with clinical features to establish a comprehensive prognostic index (ICPI).

**Results:**

Our analysis identified 90 immune cell types that were particularly relevant to BLCA. Then we constructed and validated the IRPS, with high IRPS significantly associated with longer overall survival (HR = 0.73, 95% CI, 0.71–0.76, *p* < 0.001). In two independent immunotherapy cohorts (IMvigor210 and GSE78220), patients with high IRPS demonstrated significantly prolonged survival following immune checkpoint inhibitor treatment (*p* = 0.035, *p* = 0.019). Several candidate drugs targeting IRPS were identified. The ICPI, developed by integrating IRPS with clinical features, also demonstrated enhanced accuracy in prognostic analysis.

**Conclusions:**

This study successfully developed and validated a prognostic signature (IRPS) based on comprehensive immune cell infiltration analysis, along with its integrated index (ICPI). IRPS/ICPI serves as an effective tool for predicting BLCA patient prognosis and guiding immunotherapy strategies, while also aiding in the identification of patient populations likely to benefit from immunotherapy.

## 1 Introduction

Bladder Cancer (BC) is one of the most common malignant tumors of the urinary system and ranks among the top ten prevalent cancer types globally [1]. Across these pathological types [2], bladder urothelial carcinoma (BLCA) is the most common type of bladder cancer, accounting for over 90% of cases. Based on the prediction of the American Cancer Society for 2022, there were approximately 81,180 new cases of BC diagnosed and 17,100 deaths from BC in the United States [3]. Although surgery and chemotherapy remain the primary treatment modalities, the prognosis for patients with advanced‐stage disease remains poor. The 5 year survival rate is 77%, with the 5 year survival rate for advanced BC being only 38% and 6% when the cancer has metastasized [4, 5].

Immunotherapy is a promising therapeutic method that utilizes the body's immune system to fight cancer [6]. Although checkpoint inhibitors (CPIs) have been reported to benefit cancer patients, immunotherapy still faces challenges [7]. For instance, the clinical response rate to drugs targeting PD–1/PD–L1 is limited, with only a subset of patients achieving durable benefit. Therefore, it is essential to identify the immune cell types responsible for CPI treatment and improve the efficacy of immunotherapy [7, 8].

Immunocytes (including T cells, B cells, macrophages, etc.) are major components of antitumor immune responses [9]. Recent research has demonstrated that immunocytes play significant roles in antitumor immune reactions and are related to cancer prognosis [10, 11, 12]. While some researchers have studied B‐cell behavior and sampling bias as an indicator of anti‐PD–L1 response in bladder cancer, a study focusing on all immune cell types in the tumor microenvironment (TME) remains necessary [13].

This study integrated large‐scale BLCA cohorts from multiple public databases to systematically analyze the infiltration levels of 170 immune cell features. Subsequently, a comprehensive immune cell‐related prognostic signature (IRPS) was constructed, and its value was validated across multiple dimensions, including its ability to predict patient survival, its correlation with immune microenvironment characteristics (such as immune checkpoints and stromal scores), and its predictive performance for response to ICIs therapy. Finally, by incorporating key clinical variables, a more robust integrated clinical prognostic index (ICPI) was developed. This study aims to provide effective tools for monitoring immunotherapy efficacy and predicting prognosis in BLCA patients.

## 2 Methods

### 2.1 Data Collection and Preprocessing

Figure S1 showed the details and how we constructed and validated this immune cell signature in the present study. Transcriptomic data and clinical information for BLCA patient cohorts were collected from public data sources, containing ArrayExpress (*n* = 2), the Cancer Genome Atlas (TCGA) databases (*n* = 1), gene expression omnibus (GEO) database (*n* = 10), and an immunotherapy dataset called IMvigor210 (*n* = 1). All cohorts included more than 30 samples with corresponding overall survival (OS) information. Totally 14 BLCA cohorts that met these criteria were contained in the present research (Relevant details are provided in Table S1).

Preprocessing was performed on data from different platforms. TCGA‐BLCA and IMvigor210 Datasets (http://research‐pub.Gene.com/imvigor210corebiologies) were normalized and transformed using R package “DEseq.2” [14]. Via R package “affy” [15], the raw CEL files of GEO datasets platformed on Affymetrix were first collected and further normalized by applying the robust multichip average (RMA) algorithm. The normalized expression profiles for the rest of the cohorts were retrieved directly from the related databases. From the 14 collected cohorts, the meta‐entire cohort was generated by “sva” through data preparation, incorporating and ComBat‐adjusted handling [16]. Totally, the meta‐entire cohort contained 2302 BLCA patients, 2066 of whom had complete overall survival (OS) information. All data were derived from TCGA, GEO, ArrayExpress databases, and IMvigor210 datasets, which were publicly available and had passed ethical reviews by local institutions.

### 2.2 Establishment and Prognostic Validation of the Immune Cell Related Prognostic Signature (IRPS)

After an extensive literature search on the website [17, 18, 19, 20, 21, 22, 23, 24, 25, 26, 27, 28, 29, 30, 31, 32, 33, 34, 35, 36, 37], a total of 170 immune cell types or related signatures were included in this study. The infiltration levels of immune cell signatures were explored using the single‐sample gene set enrichment analysis (ssGSEA) algorithm [38]. In this study, the infiltration level of immune cell‐related signatures was defined as the normalized enrichment score (NES).

Univariate Cox proportional hazards regression analysis was performed in each of the 14 BLCA cohorts to quantify the impact of the 170 immune cell signatures. Furthermore, a fixed‐effects model meta‐analysis was conducted using the R package “meta” [39] to obtain the pooled hazard ratio (HR) for each immune cell signature. Immune cell signatures with a meta‐analysis *p*‐value < 0.05 were defined as prognosis‐related signatures (IRPS). The IRPS was calculated using the following formula:

$$IRPS=\sum_{a=1}^{b}\text{NESa}-\sum_{c=1}^{d}\text{NESc}$$

Signatures with HR < 1 were considered protective factors, with higher NES contributing positively to the score; signatures with HR > 1 were considered risk factors, with higher NES contributing negatively to the score. Finally, based on the median NES value of BLCA samples, patients were stratified into low‐IRPS and high‐IRPS groups.

Via R package “survival”, we explored the prognostic role of the IRPS in meta‐entire and TCGA‐BLCA cohorts by survival analysis achieved between two risk groups (low, high). *p* value < 0.05 in Log‐rank test was thought significant.

### 2.3 Association of IRPS With Tumor Biological Characteristics and Immune Related Features

To elucidate the biological implications of IRPS, we conducted a comprehensive analysis of its relationship with key tumor microenvironment components and immune‐related features. The immune cell signatures' infiltration levels were compared between the two risk groups. Cancer associated fibroblasts (CAFs), accounting for more than 50% of stromal cells in tumor tissue, build a good environment for the development of tumor. So, we measured the CAFs level in BLCA patients by ssGSEA to explore the difference in CAFs among the IRPS‐risk groups. In the immune response, innate immune gene induction could be controlled by STING; we explored the correlation of IRPS with STING. Additionally, we also collected several essential immune checkpoint molecules, including CTLA4, PD–1, and PD–L1. We measured the correlation between these indices and IRPS by Pearson correlation coefficient. And the Wilcoxon test was used to compare differences between groups.

Subsequently, the “ESTIMATE” algorithm was used to calculate tumor purity, stromal score, and immune score for each BLCA sample [40]. The T cell dysfunction and exclusion (TIDE) scores of BLCA samples were evaluated from http://tide.dfci.harvard.edu/, which was used for the response of immunity treatment. What's more, Thorsson et al. defined 6 immune‐related subtypes (C1–C6) based on the pan‐cancer analysis of immune subtypes [41]. Then the differences between the IRPS and these subtypes were analyzed. Thorsson et al. also defined 56 immune‐related molecular signatures [41]. The association of IRPS with these signatures was explored. Besides, we analyzed the relationships between IRPS and immunogenic cell death (ICD) and immune checkpoint (ICP) modulators because of the important significance of these modulators in tumor immunization.

### 2.4 Exploration of the Relationship Between IRPS and the Prediction of Immunotherapy Response in BLCA

To analyze the association between IRPS and immunotherapy‐predicted pathways, we collected some signatures positively related to response to anti‐PD–L1 agent (atezolizumab) from Mariathasan's study [42, 43]. Besides, we also collected several gene characteristics related to radiotherapy response prediction and targeted therapy to explore the relationship between IRPS and these signatures.

We further evaluated the predictive value of IRPS in two independent public immunotherapy cohorts. The IMvigor210 cohort comprised 192 BLCA patients treated with atezolizumab, containing immunotherapy and survival information. The GSE78220 cohort comprised 27 patients treated with pembrolizumab, containing immunotherapy and survival information. R package “limma” was used to normalize the IMvigor210 and GSE78220 cohorts. The survival difference among the IRPS risk groups was analyzed by R package “survival”. The Kruskal–Wallis test was used to explore the IRPS difference among different response groups. Moreover, the prediction values of IRPS for immunotherapy response were explored via the receiver operating characteristic (ROC) curves and the area under the curve (AUC) [44].

### 2.5 Screening of Potential Therapeutic Drugs

To provide several new options for the therapeutic drugs in BLCA, differentially‐expressed genes (DEGs) between IRPS risk groups were first identified in the meta‐entire cohort via R package “limma”. Adjusted *p* value < 0.05 and |log2FC| ≥ 2 were the cut‐off standards for DEG identification. Similarly, we also obtained the DEGs between normal and BLCA samples. Then the up‐regulated and down‐regulated DEGs overlapping between IRPS‐related DEGs and normal‐BLCA‐related DEGs were screened out for Connectivity map (CMap) analysis via the ConnectivityMap module in CLUE (https://clue.io/). Small molecule drugs with |score| ≥ 90 were regarded as potential drugs in the present study. The mechanisms of the potential drugs were also explored by CMap mode‐of‐action (MoA) analysis. Furthermore, we conducted enrichment analyses, including Gene Ontology (GO) and Kyoto Encyclopedia of Genes and Genomes (KEGG) analyses, of these DEGs to gain a deeper understanding of the roles played by IRPS.

### 2.6 Establishing and Verifying of a Combined IRPS and Clinical Prognostic Index (ICPI)

The single‐nucleotide variants (SNV) data were downloaded from TCGA database. We explored BLCA mutation landscape using R package “maftools” [45]. The Masked Copy Number Segment (MCNS) data were downloaded via R package “TCGAbiolinks” [46]. Clinical factors between low‐ and high‐risk groups were compared, including age, gender, stage, and grade. Fisher's exact test was applied for measuring the statistical differences, with a *p* value < 0.05 considered statistically significant. In the entire meta‐cohort, the IRPS score was incorporated along with key clinicopathological features (age, sex, tumor stage, and grade) into a multivariate Cox proportional hazards model, which was subsequently validated using TCGA‐BLCA data. Variables with independent prognostic significance (*p* < 0.05) were selected to form the integrated clinical prognostic index (ICPI). The ICPI was compared with previously published prognostic gene signatures, including 3–genes signature [47], 6–genes signature [48], and 12–genes signature [49] to analyze the difference of the prognostic accuracy. Comparing standards were based on the concordance index (C–index) and the restricted mean survival (RMS) [50] curve.

## 3 Results

### 3.1 IRPS Construction and Its Role in Prognosis

**FIGURE 1:**
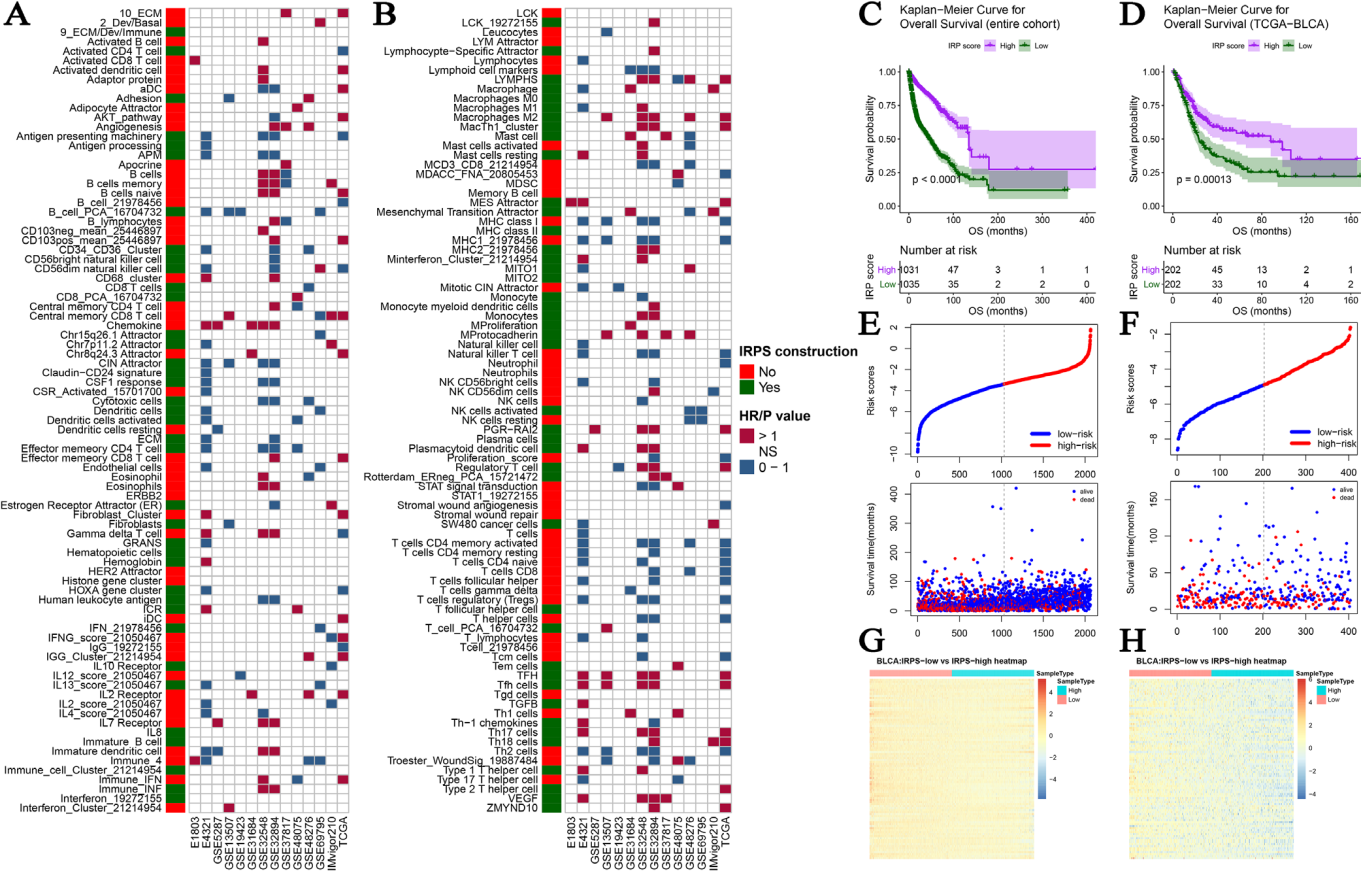
Construction of immune cell related prognostic signature/score (IRPS) and its survival role in BLCA. (A, B) Heatmap showing the prognostic value of the 170 immune types estimated by the univariate Cox model in 14 BLCA cohorts. Kalan–Meier curve for overall survival using meta‐entire cohort (C) and TCGA‐BLCA data (D). Distribution of BLCA patients in meta‐entire cohort (E) and TCGA‐BLCA data (F). (G) Heatmap of NES from 170 immune cell types for BLCA patients from meta‐entire cohort (G) and TCGA‐BLCA data (H).

This study included 170 types of immune cells. First, we evaluated the NES in over 2000 patients with BLCA from 14 publicly available cohorts (Table S2). To screen for immune cell types associated with prognosis among 170 immune cell types, we performed univariate Cox regression analysis in each of the 14 independent cohorts (Figure 1A,B). The results revealed that different immune cell types exhibited different prognostic roles. Some, such as CD4+ memory‐activated T cells and regulatory T cells, demonstrated protective effects, while TFH and Tfh cells were associated with risk effects (Figure 1A,B). Subsequently, a meta–analysis was performed on the results from the 14 cohorts to evaluate the overall prognostic value of the immune cell types. Finally, 90 immune cell types with significant prognostic value were identified (*p* < 0.05), consisting of 83 risk factors (HR > 1) and 7 protective factors (HR < 1) (Table S3). These were subsequently used to construct the IRPS. Risk factors with HR > 1 (e.g., activated B cells, activated CD4+ T cells) indicated that high NES values were associated with worse survival, whereas protective factors with HR < 1 (e.g., Th1 chemokines, IL7 receptor) indicated that low NES values were associated with better survival (Figure S2).

**FIGURE 2:**
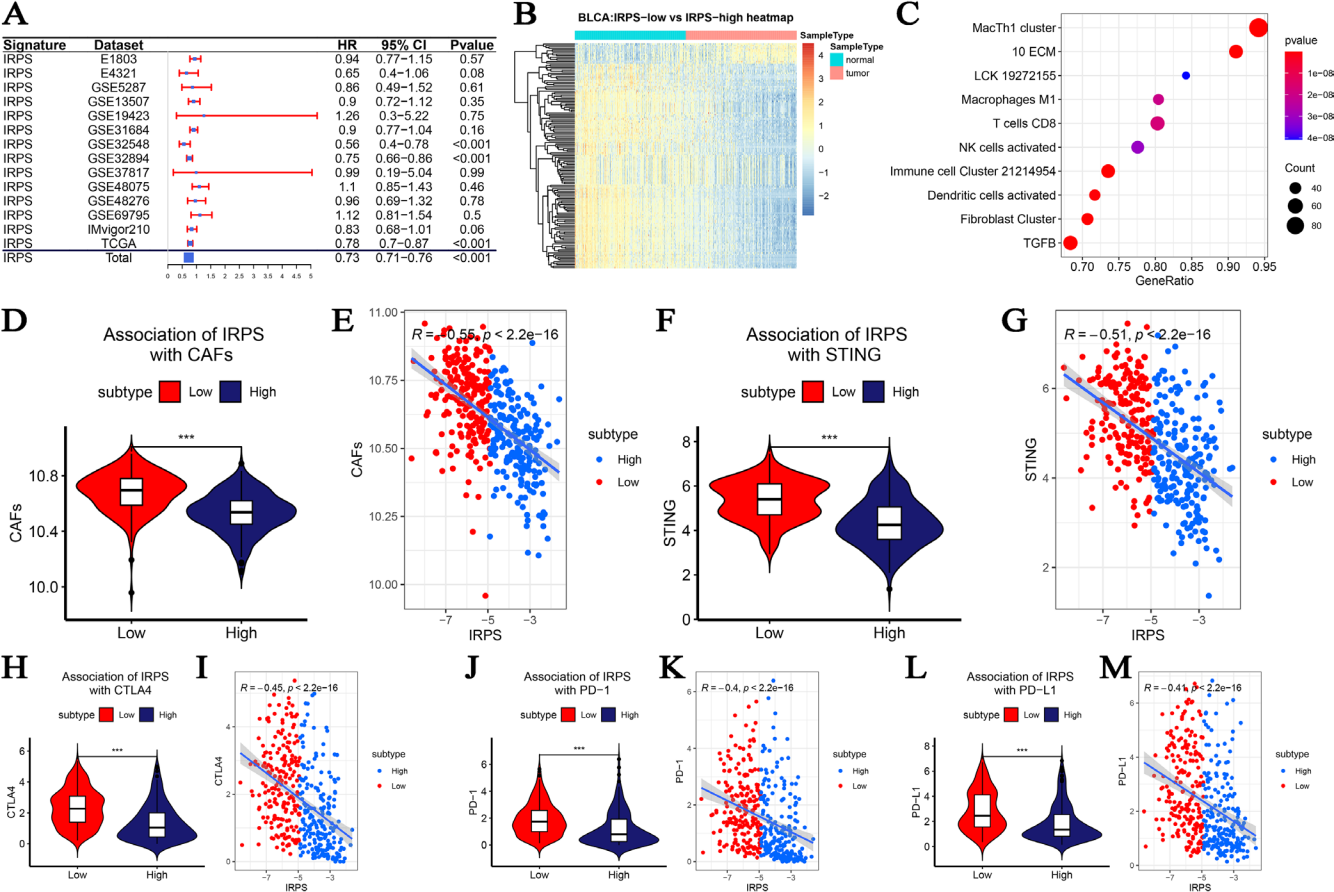
Exploration of the role of the IRPS in TCGA‐BLCA data. (A) Forest plot illustrated the HRs of overall survival analysis of the high IRPS subtype versus the low IRPS subtype in 14 BLCA cohorts. (B) differentially‐expressed gene (DEG) identification between IRPS‐high group and IRPS‐low group. (C) Enrichment analysis for these IRPS‐related genes. (D, E) The association between IRPS and cancer associated fibroblasts (CAFs) via TCGA‐BLCA data. The violin plots of CAFs for two IRPS subtypes in TCGA patients. (F, G) The association between IRPS and STING via TCGA‐BLCA data. The violin plots of STING for two IRPS subtypes in TCGA patients. (H, I) The association between IRPS and CTLA4 via TCGA‐BLCA data. The violin plots of CTLA4 for two IRPS subtypes in TCGA patients. (J, K) The association between IRPS and PD–1 via TCGA‐BLCA data. The violin plots of PD‐1 for two IRPS subtypes in TCGA patients. (L, M) The association between IRPS and PD–L1 via TCGA‐BLCA data. The violin plots of PD–L1 for two IRPS subtypes in TCGA patients.

Then, we measured the prognostic value of the IRPS using the meta entire cohort and the TCGA‐BLCA cohort. The results showed that in the meta entire cohort, BLCA patients with a high IRPS had significantly better overall survival than those with a low IRPS (*p* < 0.0001, Figure 1C). The conclusion was also validated via the TCGA‐BLCA cohort (*p* = 0.00013, Figure 1D). Analysis of the distribution of BLCA patients in the overall cohort revealed that the proportion of deceased patients was higher in the low IRPS group (Figure 1E). We concluded the similar finding via the TCGA‐BLCA cohort (Figure 1F). In addition, in BLCA patients with low IRPS, the NES levels of the 83 risk factors (immune‐related features) were higher than those in the high IRPS group (Figure 1G,H). To further confirm the prognostic value of the IRPS, we conducted univariable Cox regression analysis to analyze the overall survival difference among the 14 collected BLCA cohorts. The overall survival differences were determined across 5 of the 14 BLCA cohorts. Further meta‐analysis revealed that the IRPS was a favorable prognostic factor (HR = 0.73, 95% CI, 0.71–0.76, *p* < 0.001, Figure 2A).

We performed differentially‐expressed gene (DEG) analysis between the IRPS‐high group and IRPS‐low group (Figure 2B) with Totally 153 DEGs including 139 overexpressed genes and 14 down‐expressed genes in the IRPS‐low group (Table S4, Figure 2B). The GSEA results suggested that these IRPS‐related genes were enriched in 72 immune cell types (Table S5), such as CD8 T cells, activated NK cells, and M1 macrophages (Figure 2C). We further sought to explore the associations between the IRPS and CAFs, STING levels, as well as various fundamental immune checkpoint molecules, including CTLA4, PD–1, and PD–L1. The results suggested that BLCAs with IRPS‐low risk had greater CAF levels than those with IRPS‐high risk in the TCGA‐BLCA cohort (Figure 2D, *p* < 0.001). Moreover, BLCAs converted into the IRPS‐low risk group showed improved STING level compared with patients included by IRPS‐high risk set (Figure 2F, *p* < 0.001), and the IRPS showed a negative correlation to STING (*p* < 0.001, Figure 2G). Furthermore, BLCA patients classified into the low IRPS group exhibited significantly higher expression of CTLA4 (Figure 2H, *p* < 0.001), PD–1 (Figure 2J, *p* < 0.001), and PD‐L1 (Figure 2L, *p* < 0.001). The IRPS tended to be negatively correlated with CTLA4 (*p* < 0.001, Figure 2I), PD–1 (*p* < 0.001, Figure 2K), and PD‐L1 (*p* < 0.001, Figure 2M).

### 3.2 The Association Between IRPS With Tumor Biological Characteristics and Immune Related Features

**FIGURE 3:**
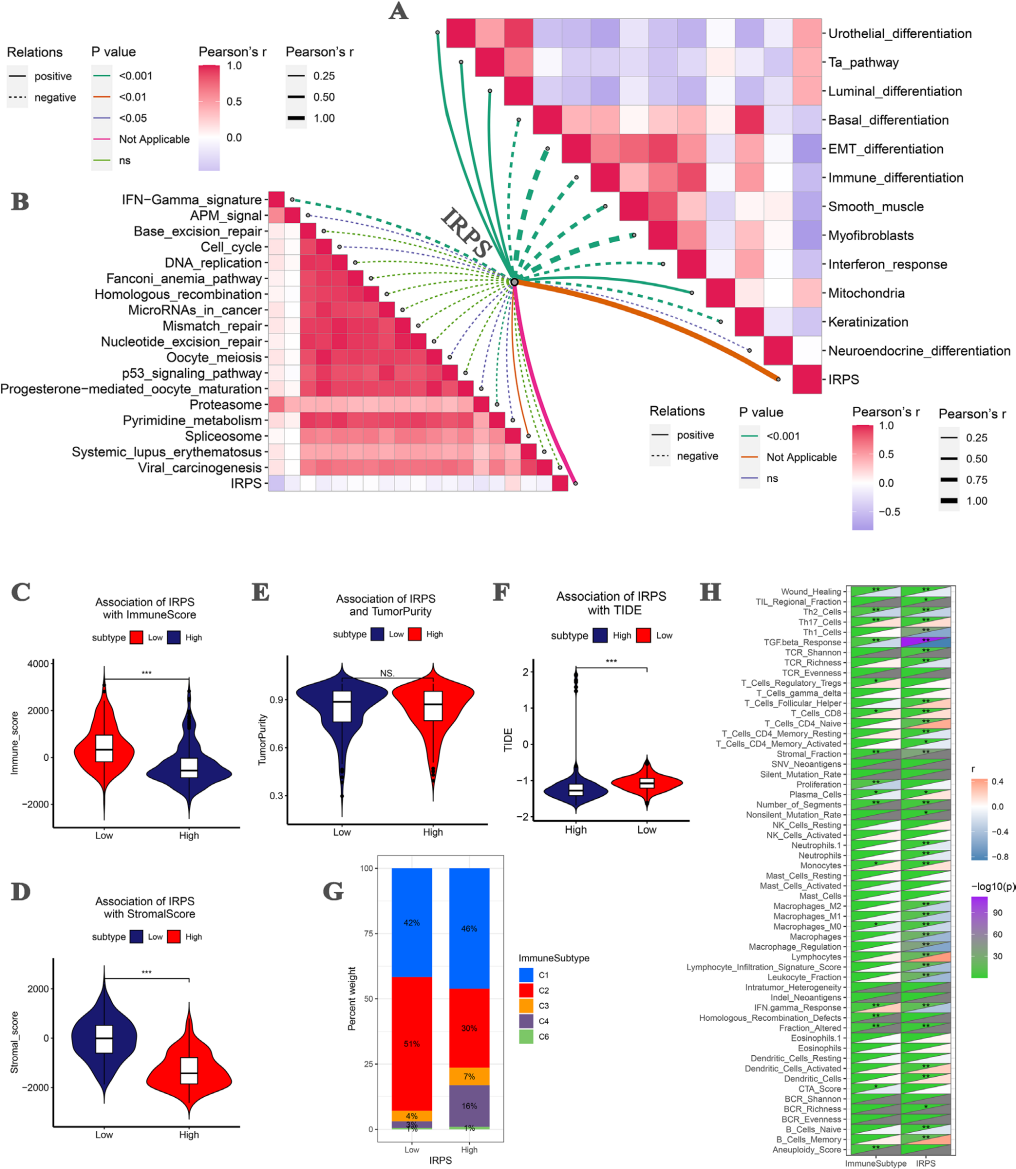
Correlation of IRPS with bladder cancer‐related pathways and immune‐related features. (A) Association of IRPS with bladder cancer‐related pathways. (B) Association of IRPS with immunotherapy response‐related pathways. (C) Association of IRPS with immune score. (D) Association of IRPS with stromal score. (E) Association of IRPS with tumor purity. (F) Association of IRPS with TIDE. (G) Association of IRPS with immune subtypes. (H) Association of IRPS with 56 molecular signatures.

To gain deeper insight into the biological significance of the IRPS, we evaluated its correlation with BLCA characteristics and immunotherapy‐related pathways. The result suggested that the IRPS was positively associated with luminal differentiation, urothelial differentiation, the Ta pathway, and mitochondria but showed a significantly negative association with interferon response, keratinization, EMT differentiation, myofibroblasts, smooth muscle, immune differentiation, basal differentiation, and neuroendocrine differentiation (Figure 3A). Furthermore, regarding immunotherapy‐related pathways, the IRPS was positively related to the spliceosome, meanwhile negatively related to IFN‐gamma signature, cell cycle, oocyte meiosis, APM signal, progesterone‐mediated oocyte maturation, and pyrimidine metabolism (Figure 3B).

To further investigate the immune microenvironment landscape of the IRPS in BLCA, the tumor microenvironment composition was evaluated using the ESTIMATE algorithm. Variability in the trends of immune and stromal scores was observed between the IRPS risk groups. Patients in the high IRPS group exhibited lower immune scores (Figure 3C, *p* < 0.001) and lower stromal scores (Figure 3D, *p* < 0.001). Furthermore, the high IRPS group had lower TIDE scores (Figure 3F, *p* < 0.001), suggesting that these patients may be less prone to immune escape and thus more likely to benefit from ICI treatment. Immuno‐subtyping analysis based on BLCA further supported this conclusion. The low risk of BLCA patients mainly overlapped with C1 and C2, which are associated with poorer prognosis. Over half of these patients were categorized into C2. Meanwhile, 16% of patients in the IRPS‐high risk set were categorized into C4, and a larger number of BLCAs with IRPS‐high risk were classified into C3, which are associated with better prognosis (Figure 3G). Single‐cell level feature analysis revealed that the IRPS was positively related to CD4+ innate T cells, CD8+ T cells, Th17 cells, and follicular helper T cells, while being negatively correlated with IFN‐γ response, macrophages, leukocyte fraction, and TGF‐β response (Figure 3H).

A further systematic analysis was conducted to examine the relationship between the IRPS and immune checkpoint molecules (ICPs). The results suggested that the IRPS was negatively related to most of the ICPs including TNFSF4, CTLA4 (which was consistent with Figure 2I), PDCD1 (PD–1, which was consistent with Figure 2K), and CD274 (PD–L1, which was consistent with Figure 2M). ICPs including TNFSF15, TNFRSF25, TMIGD2, and BTNL2 were positively correlated to IRPS (Figure S3A). In addition, the relationships between IRPS and ICD modulators were explored. EIF2AK3, EIF2AK1, and EIF2A were positively associated with IRPS, while P2RX7, LRP1, and FPR1 were negatively correlated with IRPS (Figure S3B).

### 3.3 IRPS Predicts Response to Immune Checkpoint Inhibitor Therapy

**FIGURE 4:**
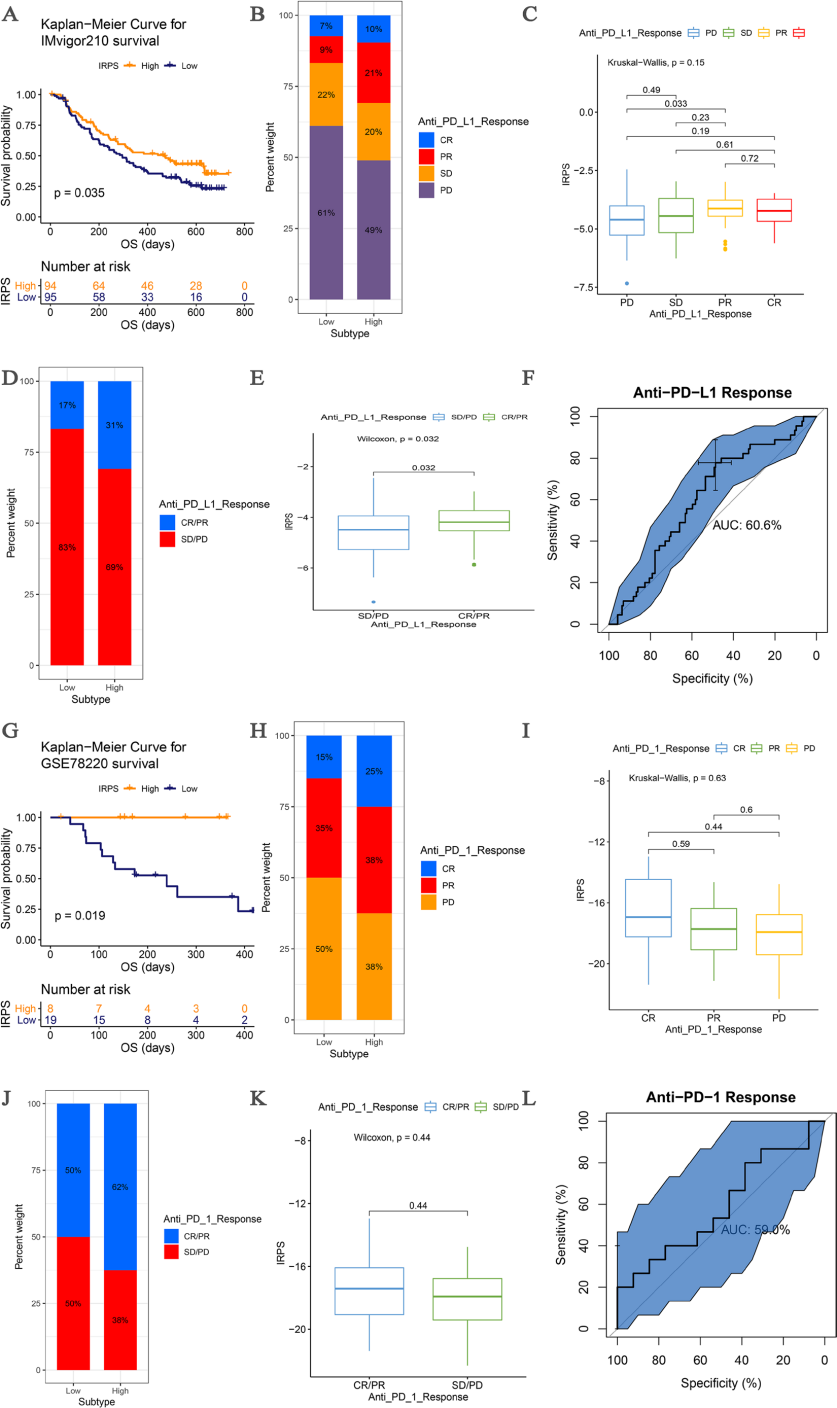
IRPS is a prognostic biomarker and predicts immunotherapeutic benefit. (A) Kaplan–Meier curves for patients with high (*n* = 125) and low (*n* = 67) IRPS in the IMvigor210 cohort. (B) Rate of clinical response (complete response (CR)/partial response (PR) and stable disease (SD)/progressive disease (PD)) to anti‐PD–L1 immunotherapy in high or low IRPS groups in the IMvigor210 cohort. (C) Distribution of IRPS in groups with different anti‐PD–L1 clinical response statuses. (D) Rate of clinical response (complete response (CR), partial response (PR), stable disease (SD) and progressive disease (PD)) to anti‐PD–L1 immunotherapy in high or low IRPS groups in the IMvigor210 cohort. (E) Distribution of IRPS in groups with different anti‐PD–L1 clinical response statuses. (F) ROC curve measuring the predictive value of the IRPS. (G) Kaplan–Meier curves for patients with high (*n* = 10) and low (*n* = 17) IRPS in the GSE78220 cohort. (H) Rate of clinical response (complete response (CR)/partial response (PR) and stable disease (SD)/progressive disease (PD)) to anti‐PD–1 immunotherapy in high or low IRPS groups in the GSE78220 cohort. (I) Distribution of IRPS in groups with different anti‐PD–1 clinical response statuses. (J) Rate of clinical response (complete response (CR), partial response (PR), stable disease (SD) and progressive disease (PD)) to anti‐PD–1 immunotherapy in high or low IRPS groups in the GSE78220 cohort. (K) Distribution of IRPS in groups with different anti‐PD–1 clinical response statuses. (L) ROC curve measuring the predictive value of the IRPS.

The predictive value of the IRPS was evaluated in two independent immunotherapy cohorts. In the IMvigor210 cohort (atezolizumab‐treated), compared with the patients with IRPS‐low risk (*n* = 95), the patients with IRPS‐high risk (*n* = 94) occupied meaningful longer survival in IMvigor210 (*p* = 0.035, Figure 4A) and patients who responded to treatment (CR/PR) had higher IRPS scores than those who did not respond (SD/PD) (Figure 4B–E). It showed that BLCA patients with a high‐risk IRPS may be more suitable candidates for anti‐PD–L1 therapy. (Figure 4B–E). The AUC of the IRPS for predicting treatment response was 0.606 (Figure 4F), indicating that the IRPS can be considered an effective tool for predicting benefit from immunotherapy. Furthermore, in the GSE78220 cohort (pembrolizumab‐treated), we observed a consistent trend. Patients with high IRPS had better survival in the GSE78220 cohort (*p* = 0.019, Figure 4G), and BLCA patients with a high IRPS were more likely to benefit from anti‐PD‐1 immunotherapy (Figure 4H–K). Similarly, the IRPS demonstrated predictive capacity for immunotherapy response (AUC = 0.590, Figure 4L). These results indicate that the IRPS is associated with response to anti‐PD–L1/PD–1 immunotherapy and that it may help predict whether BLCA patients are likely to benefit from immune checkpoint inhibitor treatment.

### 3.4 Identification of Novel Candidate Drugs Targeting IRPS

**FIGURE 5:**
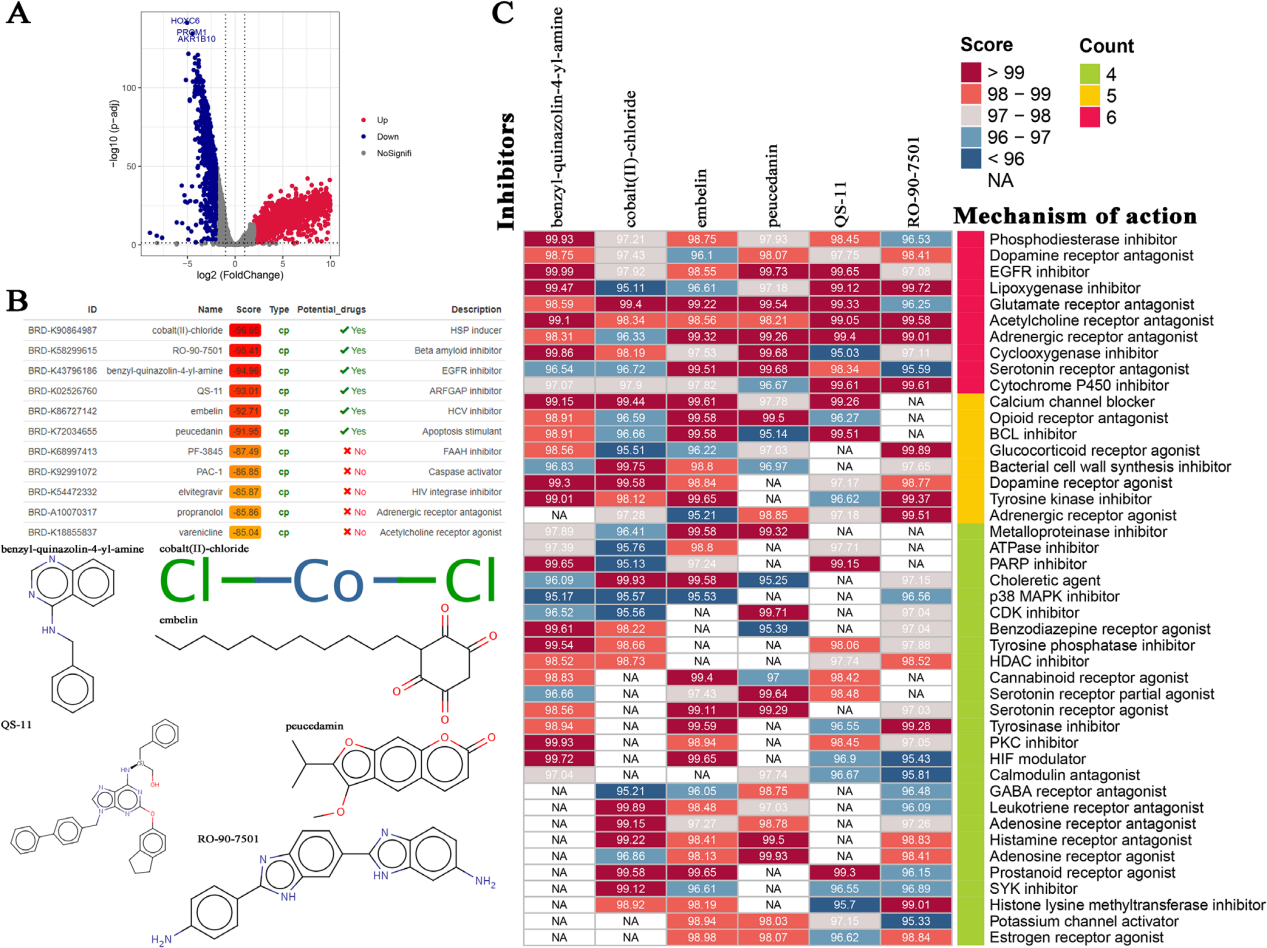
Candidate drugs targeting IRPS identification. (A) DEG identification among IRPS‐risk groups. (B) Connectivity map (CMap) analysis. (C) CMap mode‐of‐action (MoA) analysis.

To explore therapeutic strategies targeting IRPS‐related pathways, after categorizing BLCA patients into IRPS‐high and IRPS‐low risk groups, a total of 3718 DEGs containing 2124 over‐expressed DEGs and 1594 low‐expressed DEGs were determined via meta‐entire cohort (Figure 5A, Table S6). Further GO and KEGG enrichment analyses were performed on these DEGs (Figure S4). Based on the GO Biological process analysis (Figure S4A), the results revealed that IRPS was significantly correlated with positive regulation of many cell adhesion related pathways including leukocyte migration, cell chemotaxis and leukocyte chemotaxis (Figure S4A). KEGG pathway analysis revealed that IRPS was significantly correlated with many T cell related pathways including human T‐cell leukemia virus 1 infection, T cell receptor signaling pathway, and Pl3K‐Akt signaling pathway. Subsequently, CMap analysis was performed on the top 50 upregulated and 50 downregulated genes to screen for candidate small‐molecule drugs that might reverse the low‐IRPS phenotype. Cobalt (II)‐chloride (score = –96.65), RO‐90‐7501 (score = –95.41), benzyl‐quinazolin‐4‐yl‐amine (score = –94.96), QS‐11 (score = –93.01), embelin (score = –92.71), and peucedanin (score = –91.95) were screened as potential therapeutic drugs (Figure 5B). Further MoA analysis revealed that the aforementioned drugs exhibited 44 distinct mechanisms (Figure 5C). The results showed that all six drugs shared the MoA of serotonin receptor agonist, cytochrome P450 inhibitor, cyclooxygenase inhibitor, phosphodiesterase inhibitor, lipoxygenase inhibitor, adrenergic receptor antagonist, dopamine receptor antagonist, glutamate receptor antagonist, acetylcholine receptor antagonist, and EGFR inhibitor (Figure 5C).

### 3.5 Development and Validation of an Integrated Clinical Prognostic Index (ICPI)

**FIGURE 6:**
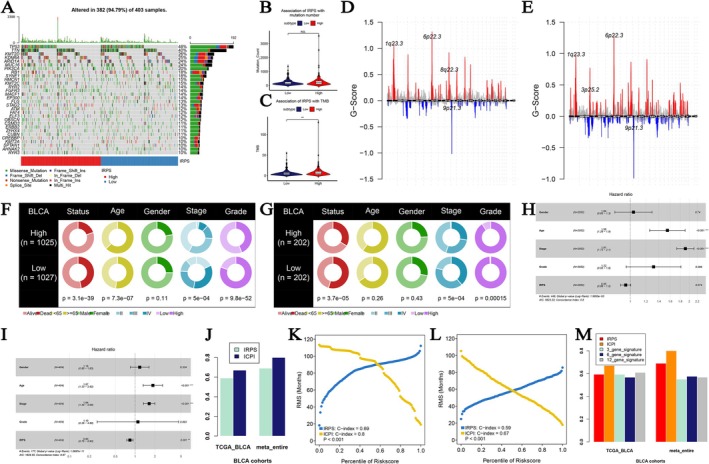
(A) The oncoplot of the top 30 mutated genes which were associated with IRPS. (B) Association of IRPS with TMB. (C) Association of IRPS with mutation number. (D) G‐score distribution among the IRPS‐high risk group. (E) G‐score distribution among the IRPS‐low risk group. The differences of clinical features (living status, age, gender, stage, and grade) across IRPS‐risk groups via meta‐entire cohort (F) and TCGA‐BLCA cohort (G). Forest plot for the Hazard Ratios (HRs) of high vs. low IRPS risk groups via meta‐entire cohort (H) and TCGA‐BLCA data (I). C‐index comparison between IRPS and ICPI (J). Restricted mean survival (RMS) curves for continuous IRPS and ICPI in meta‐entire cohort (K) and TCGA‐BLCA cohort (L) C‐index comparison between IRPS, 3–gene signature, 6–gene signature, and 12–gene signature (M).

Through a systematic analysis of the relationships between the IRPS and genomic features as well as clinicopathological factors, an integrated clinical prognostic index (ICPI) was developed. First, the relationship between the IRPS and tumor genomic features was explored. It is known that higher somatic mutation rates and higher tumor mutation burden (TMB) are related to stronger anti‐cancer immunity. Analysis revealed that the BLCAs in the IRPS‐high risk set had significantly more mutated genes (Figure 6B), and had significantly higher TMB compared to the low‐risk group (*p* < 0.01, Figure 6C). The mutation landscape was shown in Figure 6A by using the TCGA‐BLCA cohort. Besides, G‐score analysis also indicated that the patients with IRPS‐high risk had a more frequent mutation rate (Figure 6D,E). The clinical difference among these groups was explored and shown in TableS7. Regarding clinicopathological characteristics, meta‐analysis of the overall cohort revealed that IRPS high risk BLCA was significantly associated with lower stage (*p* < 0.001) and lower grade (*p* < 0.001) (Figure 6F). This finding was validated in the TCGA‐BLCA cohort (Figure 6G). There were no significant differences in age or sex distribution between the two groups (meta‐cohort *p* = 0.11; TCGA‐BLCA cohort *p* = 0.43). Based on the above findings, the IRPS and clinical characteristics (age, sex, stage, and grade) were included into the multivariable Cox analysis in meta‐entire cohort (Figure 6H) and the TCGA‐BLCA cohort (Figure 6I). Age and Stage were screened out and generated with IRPS to construct a clinically related signature: ICPI. The results showed that the ICPI significantly enhanced the predictive ability for BLCA prognosis (Figure 6J). Restricted mean survival (RMS) curve analysis further validated these findings in both the meta‐cohort (Figure 6K) and the TCGA‐BLCA cohort (Figure 6L). The ICPI was compared with previously published 3–gene, 6–gene, and 12–gene signatures to assess differences in prognostic accuracy. The results demonstrated that the ICPI exhibited the best predictive performance in both the meta‐cohort and the TCGA‐BLCA cohort (Figure 6M).

## 4 Discussion

Recent studies have indicated that immune cells could affect the prognosis and immunotherapy efficacy of BLCA patients [51]. Zhang et al. developed an immune‐infiltrating cell signature for glioma patients, which also showed significantly prognostic value in BLCA [51]. Liu et al. concluded that intratumoral TIGIT CD8 T‐cell infiltration determined immune evasion and poor prognosis in muscle‐invasive bladder cancer [52]. Das et al. performed an analysis of TCGA solid tumors and concluded that higher immune scores of BLCA were related to response to immunotherapy [53]. Dyugay et al. attempted to predict anti‐PD‐L1 response in BLCA by accounting for B‐cell behavior [13]. These studies determined that quantitative evaluation of immune cells could make novel opinions for better prognosis and immunotherapy prediction in BLCA. But none of them made a comprehensive analysis for all the immune cell types in BLCA.

In this study, through an integrated analysis of 2302 BLCA cases from 14 public datasets, we systematically constructed and validated for the first time a prognostic evaluation system based on comprehensive immune cell infiltration characteristics, termed the IRPS. The infiltration levels of 170 immune cell types were quantified using the ssGSEA algorithm. Univariate Cox regression combined with fixed‐effects model meta‐analysis was employed to identify 90 immune cell features significantly associated with prognosis, leading to the development of the IRPS scoring system. Subsequent evaluation of survival and prognostic value demonstrated that patients with a high IRPS consistently exhibited significantly better overall survival, confirming the prognostic significance of this signature.

Because of the important role of CAFs in immune function and development, we then explored the relationships between CAFs and IRPS, and the result indicated that the IRPS was significantly negatively related to CAFs. CPI treatment including anti‐PD–1, anti‐PD–L1, and anti‐CTLA4 has achieved great benefits for solid cancers. We first explored the association between these inhibitors and IRPS; the IRPS showed a negative association with all three inhibitors. The study further explored the relationship between intrinsic immune escape mechanisms and the IRPS in BLCA. The IRPS was found to be associated with several immune checkpoint molecules (ICPs), including CTLA4, PDCD1, CD274, TNFSF15, TNFRSF25, TNFSF4, TMIGD2, and BTNL2, suggesting that the IRPS may serve as a biomarker for immune checkpoint blockade therapy. The negative correlation between the IRPS and multiple immune checkpoint molecules, along with its association with TIDE scores, mechanistically supports its predictive value.

The application of the IRPS in predicting response to immunotherapy was further investigated. In two independent immunotherapy cohorts, IMvigor210 and GSE78220, patients with a high IRPS demonstrated better clinical response and survival benefit from PD–1/PD–L1 inhibitor therapy. ROC curve analysis further confirmed that the IRPS is associated with response to anti‐PD–L1/anti‐PD–1 immunotherapy, suggesting that it may serve as an effective predictive tool for evaluating the efficacy of immunotherapy.

Prediction models which unite multiple clinical features with a constructed prognostic index might show better potential for prognosis prediction and therapy guidance than a single factor. Considering the unsatisfactory prognostic value of IRPS (C‐index = 0.69), we then established the ICPI comprehensively considering the IRPS, age, and stage (which was selected by multivariable Cox analysis). The ICPI significantly improved the predictive ability of prognosis in BLCA. Compared with other previously published gene signatures, the ICPI showed superior predictive performance, providing a reliable tool for clinical decision‐making.

The potential therapeutic drugs identified based on IRPS‐related gene features offer new insights for the treatment of BLCA. Thus, we also explored some candidate drugs for BLCA treatment, including Cobalt (II)‐chloride, RO‐90‐7501, benzyl‐quinazolin‐4‐yl‐amine, QS‐11, embelin, and peucedanin. These drugs may exert their effects by modulating pathways such as lipoxygenase and cytochrome P450, which play important roles in tumor immune regulation. This finding provides a theoretical basis for the development of novel combination therapeutic strategies. In summary, the IRPS and its derivative index, the ICPI, not only enable prognostic stratification and immunotherapy prediction for BLCA patients but also offer guidance for the formulation of treatment strategies. Future research should focus on the clinical validation and mechanistic exploration of these indicators to facilitate their application in clinical practice.

### 4.1 Limitations of Study

Although this study constructed a robust immune prognostic signature by integrating multi‐cohort data, there are several limitations that require attention. Firstly, all analyses were based on retrospective data from public databases; although the reliability of the results was enhanced through rigorous meta‐analysis, independent validation in prospective clinical cohorts is lacking. Secondly, the study mainly relied on bioinformatics analyses, with a lack of experimental mechanism exploration. Thirdly, the calculation of IRPS is based on whole‐transcriptome sequencing data, which may face practical challenges in clinical translation in the current clinical diagnosis and treatment environment. In addition, the predictive value of IRPS needs to be further verified in larger‐scale and more diverse clinical cohorts. The conclusions of this study mainly provide proof‐of‐concept and directions for translational research. In the future, prospective clinical trials and experimental mechanism studies are needed to promote the translation of this system into clinical applications.

## 5 Conclusions

In conclusion, this study successfully developed and validated an immune cell‐related prognostic signature (IRPS) and its integrated prognostic index (ICPI) for BLCA. IRPS effectively stratifies patient survival and demonstrates predictive value for response to ICI therapy in independent immunotherapy cohorts. The integration of clinical features into ICPI further enhances prognostic accuracy. These findings indicate that IRPS/ICPI serves as a practical tool for guiding BLCA prognosis assessment and immunotherapy decision‐making, while also providing new directions for targeted therapy development.

## Author Contributions

Conceptualization, Wei‐Bing Zhang and Sheng Li; data curation, Xin Yan and Xiaoyu Tang; investigation, Xin Yan and Linfa Guo; methodology, Xin Yan, Ziqi He and Shaoxi Liu; software, Xiaoyu Tang, Shaojie Wu and Zhilong Li; validation, Xiaoyu Tang, Tong‐Zu Liu, Hang Zheng; Visualization, Linfa Guo, Ziqi He and Shaoxi Liu; writing – original draft, Xin Yan and Xiaoyu Tang; writing – review and editing, Zhilong Li, Wei‐Bing Zhang and Sheng Li.

## Funding

The authors have nothing to report.

## Disclosure

Inclusion and Diversity: We support inclusive, diverse, and equitable conduct of research.

## Ethics Statement

Institutional Review Board Statement: This study did not involve animals, but only data from the public database mentioned above. There were no ethical issues or other conflicts of interest, as the data was open access and ethical approval had been obtained. Based on these considerations, ethical review and approval of this study were waived.

## Conflicts of Interest

The authors declare no conflicts of interest.

## Supporting information


**Figure S1:** The flow diagram of this study.
**Figure S2:** The fix‐effect model of univariable Cox regression analysis for the 170 immune cell types.
**Figure S3:** Immune landscape of IRPS in BLCA. (A) Association of IRPS with ICPs. (B) Association of IRPS with ICD modulators.
**Figure S4:** Functional annotations of the pathways IRPS may be involved in. (A) Biological behavior of GO enrichment analysis of IRPS. (B) KEGG enrichment analysis of IRPS.

**Table S1:** Basic information of 14 included BLCA cohorts.
**Table S2:** The normalized enrichment scores for the 170 immune cell types among 2302 BLCA patients.
**Table S3:** The fix‐effect model Cox analysis for the 170 immune cell types.
**Table S4:** The details about differentially‐expressed genes (DEGs) via TCGA‐BLCA cohort.
**Table S5:** Gene set enrichment analysis (GSEA) via TCGA‐BLCA cohort.
**Table S6:** The details about differentially‐expressed genes (DEGs) via meta‐entire cohort.
**Table S7:** IRPS score and clinical information for the 2052 BLCA patients.

## Data Availability

All database generated/analyzed for this study are included/have their accession numbers included in the article.
