# Supplementary figures and images for: Characterization of Immune Infiltrating Cells in Bladder Urothelial Carcinoma and Its Clinical Significance
Source: Cancer Med. 2026 Mar 24;15(3):e71737. doi: 10.1002/cam4.71737 (PMC13140323; doi:10.1002/cam4.71737)

Figure S1


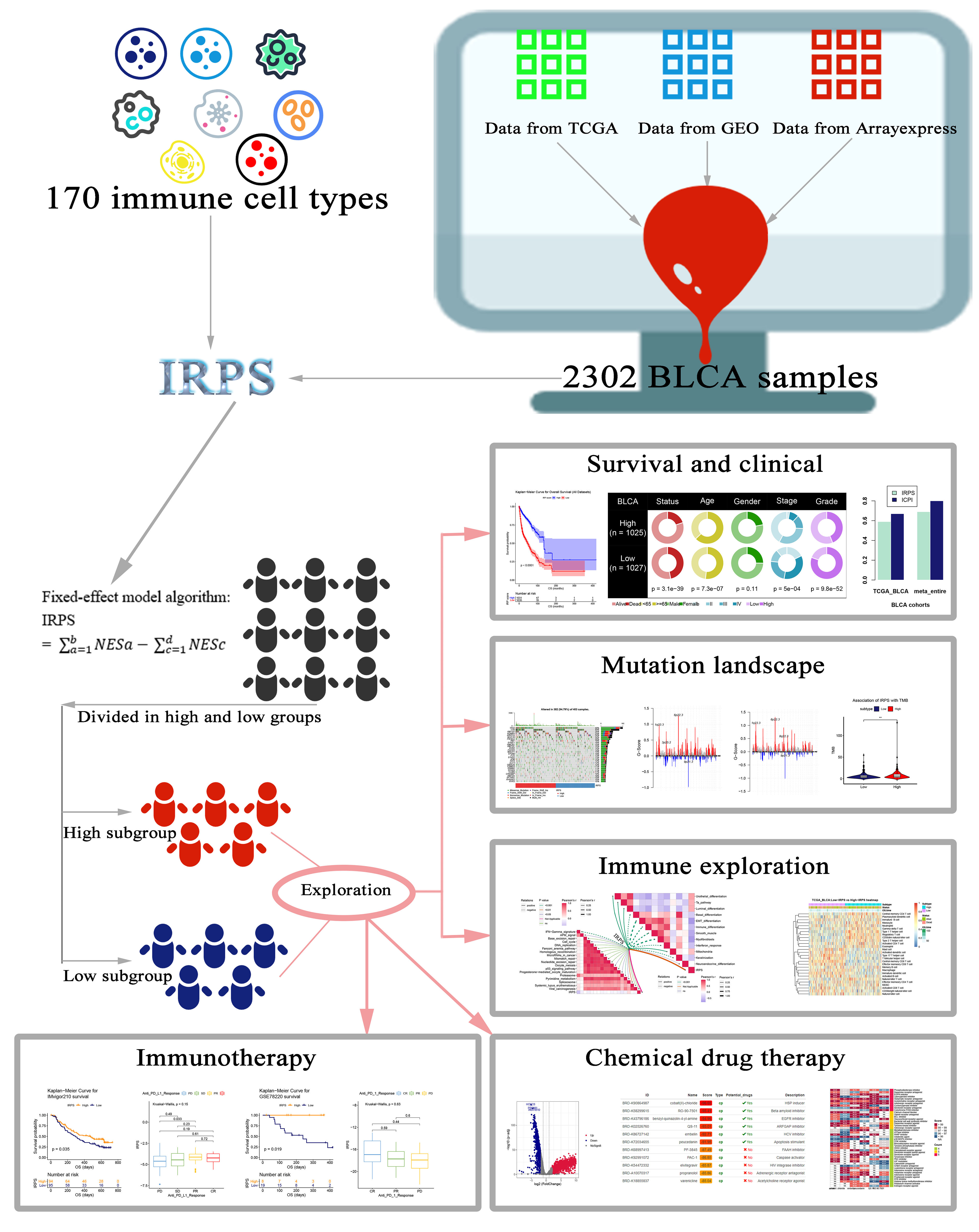


Figure S2


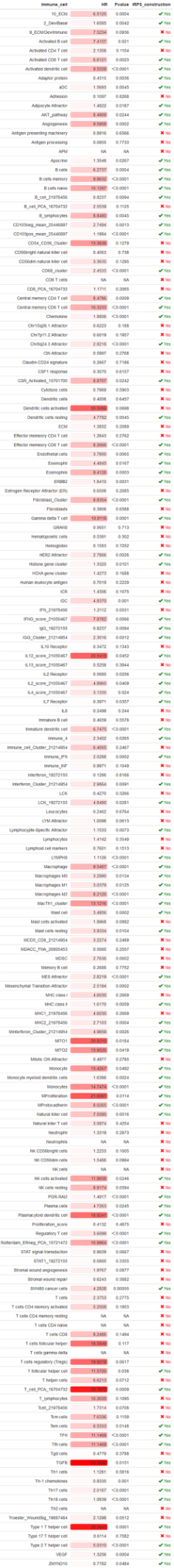


Figure S3


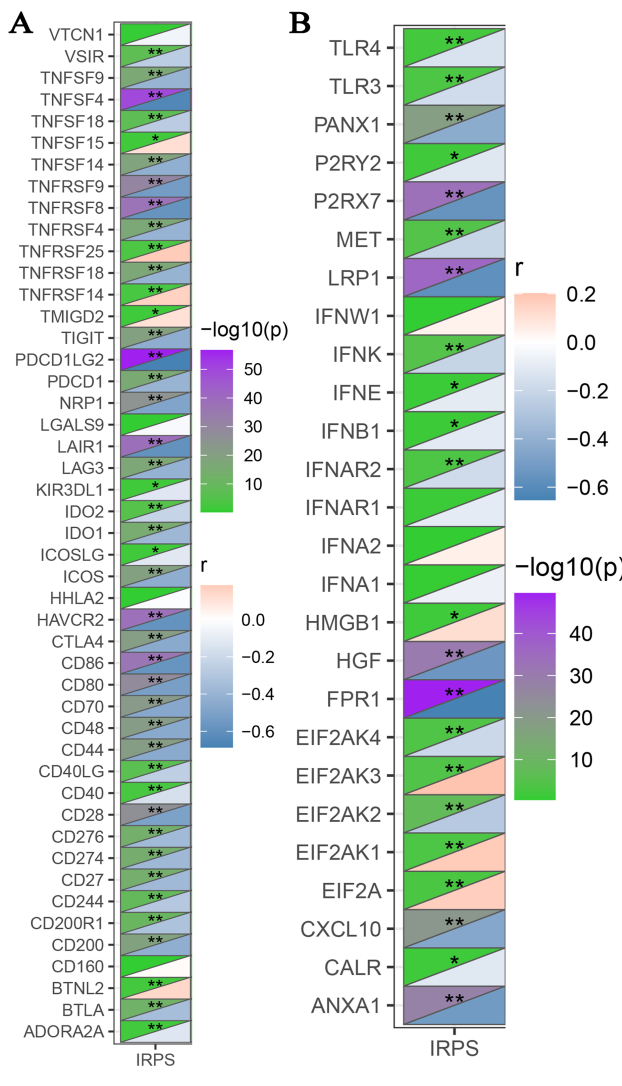


Figure S4


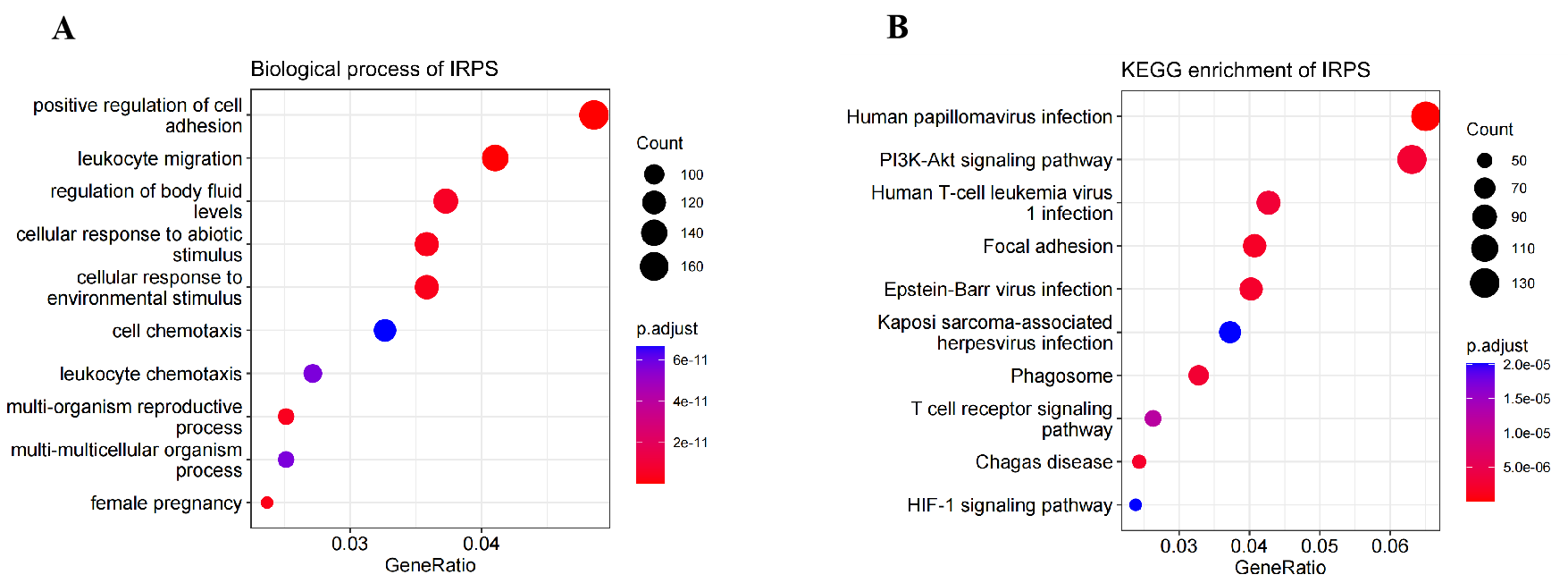

Supplement: Supplementary file 1 — Figure S1: The flow diagram of this study. Figure S2: The fix‐effect model of univariable Cox regression analysis for the 170 immune cell types. Figure S3: Immune landscape of IRPS in BLCA. (A) Association of IRPS with ICPs. (B) Association of IRPS with ICD modulators. Figure S4: Functional annotations of the pathways IRPS may be involved in. (A) Biological behavior of GO enrichment analysis of IRPS. (B) KEGG enrichment analysis of IRPS. [file CAM4-15-e71737-s002.docx]
